# Medical student and academic staff perceptions of role models: an analytical cross-sectional study

**DOI:** 10.1186/1472-6920-6-9

**Published:** 2006-02-17

**Authors:** Ali A Haghdoost, Mohammad R Shakibi

**Affiliations:** 1 Education development centre, Deputy of Education, Kerman University of Medical Sciences, Jomhoori Islami Blvd, Postal code: 7618747653, Kerman, Iran; 2 Internal Medicine Department, Afzalipoor Hospital, Kerman, Iran

## Abstract

**Background:**

This study explored the associations between the perceptions of students and the perceptions of academic staff about the characteristics of clinical lecturers at the Department of Internal Medicine at Kerman University of Medical Sciences (KUMS). It also assessed what characteristics constitute a 'role model' from the point of view of students and staff.

**Methods:**

Staff and students were questioned about the characteristics of their colleagues and lecturers, respectively. They were asked about 15 characteristics under four headings: personality, teaching skill, group working and overall performance as a role model.

Associations between lecturers' characteristics were explored using Pearson correlation and characteristics were allocated into groups by partition cluster method. In addition, predictors of being a valuable lecturer were assessed using logistic regression analysis.

**Results:**

Based on staff responses, the strongest association observed was between honesty and being respectful (r = 0.93, p < 0.0001). Based on student responses, the strongest association observed was between being professional and honesty (r = 0.98, p < 0.0001). None of the correlations between student and staff perceptions were significant for any characteristic.

Two groups were recognized among the characteristics. group one contained those characteristics which were related to the lecturer's activity; while the second group contained characteristics that were related to the personality or teaching performance of the lecturer.

The predictors of lecturer as 'role model' (i.e., perceptions of students) consisted mostly of characteristics from the first group, while the predictors of a 'role model' by fellow academic staff consisted of characteristics that were in both groups.

**Conclusion:**

These findings showed considerable differences between the perceptions of students about their lecturers when compared with perceptions of staff about their colleagues. Students were more concerned with the personality of their lecturers, while staff also considered their ideas and behaviors. This suggests that a more comprehensive assessment of a lecturer's performance could be obtained by taking into account feedback from both students and colleagues.

## Background

In addition to knowledge and skills, the personality of a lecturer plays an important role in the learning process. Exposing students to excellent role models inspires them to study better[1]. Successful lecturers communicate with students and their colleagues more effectively. Successful lecturers are also able to change the attitude and of their students as well as improve knowledge and skills. The personality of a lecturer can have a strong effect on the behavior and attitude of his students [2-4].

The role of a lecturer's personality is much more important in clinical teaching than in theoretical classes. Students spend many hours with their clinical lecturer, watch his behavior and practice and learn how to approach to patients. Since the learning process takes place in real situations during their contacts with patients in clinics and hospitals, the clinical lecturer's personality has much more influential effects on the students. The clinical lecturer simultaneously teach theoretical issues, show competence in his practices, and demonstrate his personality in direct contact with patients. [5-7].

According to the above explanation, it seems that lecturers, particularly in clinical fields, should pay more attention to their characteristics if they would like to improve the quality of their teachings. In other words, they should believe that only their knowledge and even teaching skills are not enough for an effective clinical teaching[8].

In addition, it is important to know how much the lecturers' and students' perceptions about the lecturers' characteristics are comparable. In many colleges such as KUMS, feedback from students is the main source of information to evaluate the performance of educators[9]. However, there is evidence that shows that students' feedback may be distorted by other factors such as the punctuality of educators, or even by the complexity of the courses [10-15]. In addition, some authors believe that the narrow view of students during their evaluation cannot illustrate a wide and deep view from the capacity and performance of their lecturers. Therefore, it might be useful to know how much students' perceptions are compatible with the lecturer's perceptions and in which characteristics their perceptions differ.

This study was conducted in a clinical setting to evaluate the characteristics of clinical lecturers in an internal medicine department which is the largest clinical department in KUMS. It compared students' and lecturers' perceptions and explored the main personal characteristics of successful and influential educators. Although a great deal of research has been published on this issue, there are only a few publications about the characteristics of role models from developing countries, which is an appropriate topic for further studies.

## Method and materials

The internal medicine department in KUMS has 23 clinical lecturers, with five subspecialties (gastroenterology, nephrology, rheumatology, pulmonary and infectious diseases). This department trains around 25 to 30 students every three months. Neurology and cardiology have their own departments in KUMS.

The questionnaire was designed as a grid; rows showed the lecturers' names and columns showed fifteen characteristics under four headings: personality, teaching skills, group working skills and overall performance as a role model. Thirteen of these characteristics were the same in student's and staff's questionnaires which were: punctual, knowledgeable, ethical, patient, compassionate, honesty, adept in group work, respectful, professional, capable manager, idealist, pragmatic and energetic. Two extra characteristics in staff's questionnaires were helpful friend and valuable colleague. The two distinctive characteristics in the student's questionnaires were positive role model and meritorious. These items were mostly borrowed from the formal questionnaires which are being used in KMUS and some other Medical Universities in Iran to assess the performance of academic staff; the validity of these questionnaires have been assessed in previous studies[16,17].

The questionnaires were distributed among the academic staff in their monthly meeting. At the meeting, the objectives of the study were clearly explained. The participants were allowed to ask questions and also to discuss any issues regarding the content of the questionnaire. All participants were encouraged to complete the questionnaire individually based on their best knowledge about their colleagues and were instructed to tick corresponding cells only if they were confident that their colleague had any of those characteristics.

Around 50% of the staff participants completed the questionnaire during the meeting and about 50% requested more time to complete the questionnaire. These respondents were approached individually and their completed questionnaires were collected within two weeks.

All of the students were asked to complete the questionnaire during their last week of rotation in the internal medicine department using the same guidelines as specified above for staff.

Having checked the normality of the variables with one sample Kolmogorov-Smirnov test, the correlation between the responses of students and staff were assessed using Pearson correlation coefficient. Using partition cluster analysis, the potential groups among these characteristics were assessed. To check the accuracy of the generated clusters, the Minkowski Euclidean Distance was computed.

In addition, it was assessed which of the lecturer's characteristics had the strongest links with extra characteristics in staff's questionnaires (helpful friend and valuable colleague) and student's questionnaires (positive role model and meritorious). These associations were modeled using logistic regression; the significant variables were entered in the models using conditional forward method.

The data were analyzed in Stata version 8. The maximum accepted type one error was 0.05.

## Results

Three out of twenty three academic staff in the internal medicine department were on sabbatical during the data collection period, and four staff declined participation. Responses were collected from all of the students (n = 26).

Based on both students' and staff's responses, strong correlation coefficients were observed among the characteristics (table 1). Based on the staff responses, the strongest correlation coefficient was between honesty and respectfulness (r = 0.93, p < 0.0001). Based on the student responses, the strongest correlation was between being professional and honesty (r = 0.98, p < 0.0001); the weakest correlation was between being energetic and idealistic (r = 0.27, p = 0.3).

Using cluster analysis, two groups were generated. The characteristics in group one were being energetic, pragmatic, idealistic and a capable manager; all other characteristics were included in group two. The dissimilarity index among all characteristics was 20.01, and 14.56 and 6.46 among characteristics in group one and two, respectively. The considerably lower dissimilarity indexes within both groups indicated that the characteristics were allocated between these two groups appropriately. The lower index in group two demonstrated that those characteristics were much more correlated than those in the group one. (Table 2)

None of the correlation coefficients between students' and staff's responses were significant for any of the characteristics. Nevertheless, the strongest and weakest correlations were observed in being energetic (r = 0.37) and pragmatic (r = 0.01) respectively (Table 3). The statistical power of the test for the maximum observed r was 0.78. This suggested that student perceptions about their lecturers' characteristics were not the same as staff perceptions about their colleagues.

According to the staff responses, the predictors of the helpful friend and valuable colleague were completely different. The significant predictors for helpful friend were being ethical, a capable manager, energetic and compassionate. The significant predictors for valuable colleague were being idealistic, honesty, respectful and punctual. (Table 4)

According to the students' responses, some predictors of the being positive role model and meritorious were similar such as being respectful, compassionate, and energetic. Nonetheless, some characteristics such as being professional, honesty, and pragmatic were significant predictors of being a positive role model. On the other hand, being knowledgeable, adept in group work and a capable manager were significant predictors of being meritorious (Table 4). The three strongest predictors of both of these characteristics were belonged to group two.

## Discussion

This study showed that students' perceptions about the characteristics of their lecturers were not the same as staff's perceptions about their colleagues. In addition, the staff's characteristics were summarized in two groups, the group one contained those characteristics conceptually were more related to the performance of lecturers; while the characteristics in group two were more related to the personality and teaching skills of lecturers. The results also showed that based on students' responses, the predictors of being a positive role model and meritorious were more related to characteristics in group two; while, based on staff's responses, the predictors of being helpful friend and valuable colleague were related to the characteristics of both groups.

Although it is recommended to evaluate the performance of educational system based on the students' feedback, the results of this study, compatible with many other studies, showed that there are considerable discrepancies between staff's and students' views[8,9,12,18]. Even the maximum correlation coefficient between students' and staff's perceptions was less than 0.4. In teaching skills and knowledge, which could be considered more relevant to the teaching process, the coefficients were less than 0.3 and 0.15 respectively, offering support to the theory that relying on the student feedback alone in the evaluation of a lecturer's performance may not be valid.

One of the main limitations of this study was the small sample size. The lack of statistically significant coefficients may be due to this limitation.

The deviance (dissimilarity index) among the characteristics in group two was low. Conceptually, those characteristics mostly evaluate the personality of lecturers (being punctual, ethical, patient, compassionate, honestyr, adept in group work, Respectful) or their teaching skills and knowledge. Therefore, it could be inferred that those characteristics had substantial associations and a lecturer may improve those characteristics in himself or herself simultaneously.

The predictors for being helpful friend and valuable colleague were completely different. Those predictors were also different from the predictors of being a positive role model and meritorious. Nonetheless, being respectful, compassionate and energetic were significant predictors for both being a role model and meritorious. It should be added that the knowledge and teaching skills of educators also were chosen by students as the first and second most important predictors in either of these two distinctions, which is compatible with the findings of Elzubier et. al. who reported that the personality and teaching skills of lecturers had the strongest associations with being a positive role model in clinical teaching[1]. This implies that a good and knowledgeable lecturer who is compassionate, respectful and active may have the strongest influence on students, probably in all cognitive, behavioral and affection aspects.

## Conclusion

These findings showed considerable differences between the student perceptions about their lecturers and staff perceptions about their colleagues. Therefore, relying on the student feedback alone in the evaluation of a lecturer's performance may not be valid.

In addition, students chose model and meritorious lecturers based on their lecturers' personality, knowledge and performance. While staff chose their helpful friends and valuable colleagues based on their ideas, personalities and behaviors.

## Competing interests

The author(s) declare that they have no competing interests.

## Authors' contributions

AAH designed the study, collected and analyzed the data and wrote the paper. MRS helped in designing the study, and had considerable contribution in collecting data. All authors read and approved the final manuscript.

## Pre-publication history

The pre-publication history for this paper can be accessed here:



## Figures and Tables

**Table 1:** Correlation coefficients between academic staff characteristics* (bold = student responses, unbold = staff responses)

	Punctual	Knowledgeable	Ethical	Patient	Compassionate	Honesty	Adept in group work	Respectful	Professional	Capable manager	Idealistic	Pragmatic	Energetic
Punctual		0.6	0.32	0.59	0.77	0.67	0.76	0.63	0.77	0.65	0.67	0.76	0.62
Knowledgeable	**0.91**		0.3	0.54	0.72	0.57	0.76	0.50	0.76	0.43	0.64	0.59	0.67
Ethical	**0.88**	**0.80**		0.84	0.50	0.71	0.51	0.75	0.38	0.14	0.22	0.27	0.24
Patient	**0.94**	**0.87**	**0.95**		0.76	0.87	0.72	0.83	0.60	0.33	0.46	0.44	0.53
Compassionate	**0.95**	**0.92**	**0.87**	**0.95**		0.77	0.73	0.69	0.72	0.38	0.80	0.65	0.78
Honesty	**0.94**	**0.93**	**0.92**	**0.97**	**0.95**		0.76	0.93	0.73	0.46	0.67	0.55	0.60
Adept in group work	**0.83**	**0.86**	**0.81**	**0.87**	**0.88**	**0.90**		0.77	0.80	0.57	0.62	0.67	0.59
Respectful	**0.90**	**0.88**	**0.97**	**0.96**	**0.93**	**0.96**	**0.88**		0.71	0.55	0.61	0.63	0.60
Professional	**0.96**	**0.93**	**0.91**	**0.97**	**0.95**	**0.98**	**0.88**	**0.95**		0.63	0.77	0.78	0.66
Capable manager	**0.84**	**0.82**	**0.62**	**0.80**	**0.79**	**0.83**	**0.79**	**0.71**	**0.91**		0.55	0.73	0.42
Idealistic	**0.70**	**0.63**	**0.65**	**0.70**	**0.71**	**0.61**	**0.69**	**0.68**	**0.60**	**0.40**		0.74	0.72
Pragmatic	**0.70**	**0.70**	**0.58**	**0.75**	**0.70**	**0.75**	**0.73**	**0.64**	**0.81**	**0.88**	**0.36**		0.76
Energetic	**0.46**	**0.59**	**0.58**	**0.63**	**0.54**	**0.68**	**0.67**	**0.62**	**0.57**	**0.54**	**0.27**	**0.63**	

*Underlined coefficients are significant at p < 0.05 using Bonferroni correction

**Table 2:** Characteristics allocated into two groups based on their intra-group correlations using cluster analysis

	***Characteristics***	Dissimilarity Index*
Group 1	Energetic, Pragmatic, Idealistic, Capable manager	14.55
Group 2	Punctual, Knowledgeable, Ethical, Patient Compassionate, Honesty, Adept in group work, Respectful, Professional	6.46

All	All of the above characteristics	29.01

*Using partition cluster analysis the Minkowski Euclidean Distance was computed

**Table 3:** Correlation coefficients between student and staff perceptions of lecturer characteristics

*characteristic*	Correlation Coefficient	p-value
Pragmatic	0.01	0.98
Punctual	0.05	0.83
Patient	0.09	0.7
Adept in group work	0.1	0.68
Knowledgeable	0.12	0.59
Compassionate	0.19	0.4
Idealistic	0.23	0.33
Ethical	0.27	0.24
Professional	0.27	0.23
Honesty	0.31	0.22
Respectful	0.31	0.22
Capable manager	0.31	0.21
Energetic	0.37	0.14

**Table 4:** Associations between distinctive characteristics as dependent variables and the other characteristics, classified by staff and student responses

Respondent	Characteristic	Predictors	Odds ratio (95% CI)	Adjusted R^2^*
Staff	Helpful friend	Ethical	8.6(3.1–24.1)	0.54
		Capable manager	8.1(3.1–20.1)	
		Energetic	5.4(2.1–13.6)	
		Compassionate	3.9(1.4–10.7)	
	(precious) Valuable colleague	Idealist	13.8(1.6–122.4)	0.57
		Honesty	10.7(3.3–35.1)	
		Respectful	8.1(2.5–26.3)	
		Punctual	5.0(1.7–14.6)	
Student	Positive role Model	Professional	8.6(2.6–28.7)	0.5
		Honesty	4.5(1.3–15.7)	
		Respectful	4.2(1.3–14.1)	
		Compassionate	4.1(1.5–11.2)	
		Pragmatic	3.8(1.7–8.5)	
		Energetic	3.4(1.5–7.8)	
	Meritorious	Compassionate	10.7(2.7–41.5)	0.47
		Knowledgeable	8.4(1.0–73.6)	
		Respectful	5.8(1.7–19.7)	
		Energetic	3.8(1.8–7.9)	
		Adept in group work	2.5(1.2–5.2)	
		Capable manager	2.4(1.1–4.9)	

* Cox and Snell R^2^, the variables were entered in the logistic regression model using conditional forward method
